# Understanding the multilevel determinants of clinicians’ imaging decision-making: setting the stage for de-implementation of low-value imaging

**DOI:** 10.1186/s12913-022-08600-3

**Published:** 2022-10-05

**Authors:** Soohyun Hwang, Sarah A. Birken, Matthew E. Nielsen, Jennifer Elston-Lafata, Stephanie B. Wheeler, Lisa P. Spees

**Affiliations:** 1 Department of Health Policy and Management, Gillings School of Global Public Health, The University of North Carolina at Chapel Hill, 27599-7411 Chapel Hill, NC USA; 2 Department of Implementation Science, School of Medicine, Wake Forest University, Winston-Salem, USA; 3 UNC Lineberger Comprehensive Cancer Center, The University of North Carolina at Chapel Hill, Chapel Hill, USA; 4 Department of Epidemiology, Gillings School of Global Public Health, The University of North Carolina at Chapel Hill, Chapel Hill, USA; 5 Department of Urology, School of Medicine, The University of North Carolina at Chapel Hill, Chapel Hill, USA; 6 UNC Eshelman School of Pharmacy, The University of North Carolina at Chapel Hill, Chapel Hill, USA

**Keywords:** Hematuria, Prostate cancer, Imaging, De-implementation, Low-value care, Behavior change, Mixed methods, CFIR

## Abstract

**Background:**

De-implementation requires understanding and targeting multilevel determinants of low-value care. The objective of this study was to identify multilevel determinants of imaging for prostate cancer (PCa) and asymptomatic microhematuria (AMH), two common urologic conditions that have contributed substantially to the annual spending on unnecessary imaging in the US.

**Methods:**

We used a convergent mixed-methods approach involving survey and interview data. Using a survey, we asked 33 clinicians (55% response-rate) to indicate their imaging approach to 8 clinical vignettes designed to elicit responses that would demonstrate guideline-concordant/discordant imaging practices for patients with PCa or AMH. A subset of survey respondents (N = 7) participated in semi-structured interviews guided by a combination of two frameworks that offered a comprehensive understanding of multilevel determinants. We analyzed the interviews using a directed content analysis approach and identified subthemes to better understand the differences and similarities in the imaging determinants across two clinical conditions.

**Results:**

Survey results showed that the majority of clinicians chose guideline-concordant imaging behaviors for PCa; guideline-concordant imaging intentions were more varied for AMH. Interview results informed what influenced imaging decisions and provided additional context to the varying intentions for AMH. Five subthemes touching on multiple levels were identified from the interviews: National Guidelines, Supporting Evidence and Information Exchange, Organization of the Imaging Pathways, Patients’ Clinical and Other Risk Factors, and Clinicians’ Beliefs and Experiences Regarding Imaging. Imaging decisions for both PCa and AMH were often driven by national guidelines from major professional societies. However, when clinicians felt guidelines were inadequate, they reported that their decision-making was influenced by their knowledge of recent scientific evidence, past clinical experiences, and the anticipated benefits of imaging (or not imaging) to both the patient and the clinician. In particular, clinicians referred to patients’ anxiety and uncertainty or patients’ clinical factors. For AMH patients, clinicians additionally expressed concerns regarding legal liability risk.

**Conclusion:**

Our study identified comprehensive multilevel determinants of imaging to inform development of de-implementation interventions to reduce low-value imaging, which we found useful for identifying determinants of de-implementation. De-implementation interventions should be tailored to address the contextual determinants that are specific to each clinical condition.

**Supplementary Information:**

The online version contains supplementary material available at 10.1186/s12913-022-08600-3.

## Background

Through the Choosing Wisely campaign and similar initiatives, the use of low-value care (LVC) has gained attention [1, 2] and increased recognition of the need for de-implementation in practice [3]. Inappropriate and unnecessary imaging is one type of LVC which comprises approximately $30 billion of unnecessary annual spending in the US [3]. While imaging can allow clinicians to detect and diagnose medical conditions, including cancer, earlier and more accurately [1, 2], extant research indicates that imaging often has low-value – i.e., used when not clinically meaningful. In particular, there has been a continuous effort to identify the most cost-effective strategy for the diagnostic evaluation of patients with low-risk prostate cancer (PCa) and patients with asymptomatic microhematuria (AMH) to reduce unnecessary costs and to increase diagnostic accuracy [4, 5]. Prostate cancer is the most common cancer diagnosed in men in the US, with over 250,000 men projected to be diagnosed in 2022 [6]. Despite the recommendations against low-value imaging from the Choosing Wisely campaign [7] and the ‘Less is More’ movement [8], as well as similar stances of professional societies such as American Urological Association (AUA) and American Society of Clinical Oncology (ASCO), low-value imaging remains common; up to 48% of patients with low-risk PCa receive potentially avoidable imaging [9, 10]. For instance, routine CT of the pelvis or bone scan for men with very low- or low-risk PCa may be considered low-value imaging. Similar to the context of PCa, hematuria is frequently encountered in clinical practice, with millions of patients referred to urologists for further evaluation [11]. The High Value Care Task Force of the American College of Physicians has called into question the need for intensive imaging of patients with AMH [12]. Most recently, the AUA revised their guideline for hematuria, with recommendations substantially reducing the target population for computed tomography (CT) scan [13], in a departure from prior guidance recommending CT scan for all adults with hematuria [14].

To date, studies examining the determinants of imaging often focus on patient-level factors, which are presumed to have the strongest influence on imaging decisions [15, 16]. However, a recent scoping review concluded that determinants of the use of LVC are embedded at multiple levels (e.g., reimbursement policy; providers’ beliefs about consequences of LVC, providers’ liability risk) [17]. Findings from the review also implied that strategies for reducing LVC should reflect determinants of the use of the specific low-value practice (i.e., imaging) [17]. In other words, identifying the multilevel determinants of imaging on a broader scope (not just of low-value imaging) is needed to comprehensively understand the contextual factors that contribute to clinicians’ decision-making process. Ultimately, understanding contextual determinants of imaging may inform strategies to decrease low-value imaging. Against this backdrop, the objective of this study was to identify multilevel determinants of imaging for PCa and AMH, two urologic conditions where low-value imaging is common.

## Methods

### Setting/procedure

Data were collected from April 2019 to November 2019. Eligible participants included clinicians specializing in urologic care with the authority to order imaging and currently practicing within one of three university-affiliated healthcare systems in a single state. It is typical for the clinicians specializing in urologic care to provide care (e.g., order imaging) upon referral from their primary care in the U.S. We collected participants’ contact information, including their email and phone number, from publicly available information on each health system’s website and was able to construct a list of 60 potentially eligible participants from three different health systems. If no contact information was provided, we called the listed number of the clinician’s practice to obtain the clinician’s work email. The Institutional Review Board at the University of North Carolina at Chapel Hill approved the study and waived written consent due to minimal risks to study participants.

We used a convergent mixed-methods approach in which survey and interview data were collected concurrently. This approach was utilized to assess the extent of and context surrounding guideline-discordant imaging (via surveys) and the context surrounding and influencing provider imaging behavior (via interviews). In other words, the qualitative findings complemented the quantitative survey results by adding a narrative understanding of the multilevel determinants of imaging decision-making. We emailed 60 eligible respondents an online survey (details below). At the end of the survey, we asked respondents if they would be willing to participate in a one-time telephone interview regarding their imaging practices. We contacted 10 respondents who expressed initial interest. Once an interview was completed, participants were given a $20 Amazon gift card as a token of appreciation.

### Measurements

#### Structured surveys

The survey data were collected via online self-administered surveys coordinated by Qualtrics and the answers were not anonymous. The structured survey included 8 short clinical vignettes describing patients newly diagnosed patients with either PCa or AMH. Scenarios were designed to elicit responses that would demonstrate guideline-concordant/discordant imaging practices relative to the clinical practice guidelines available from professional societies at the time of the study for each condition [14, 18]. For the PCa patient vignettes, we included patient demographic and clinical information such as age, prostate specific antigen (PSA) levels, and Gleason score. For AMH patient vignettes, we included age, smoking status, sex, and urinalysis results. Questions following each vignette inquired about the type of imaging, if any, the respondent would use. For PCa patient vignettes, respondents were asked to mark all that apply among a list that included MRI, CT scan, and bone scan; for AMH patient vignettes, the list included renal ultrasound, CT urogram, MR urogram, and flexible cystoscopy. Respondents were also able to write in a different action/procedure in free-text comment boxes, if needed. To ensure face and content validity of clinical scenarios and imaging options, two pilot surveys were conducted with practicing urologists.

#### Semi-structured interviews

To comprehensively examine the potential determinants of imaging, we employed the Consolidated Framework for Implementation Research (CFIR) and Theoretical Domains Framework (TDF; hereafter CFIR + TDF) to guide the development of the semi-structured interview. While both determinant frameworks are considered comprehensive, the TDF focuses on individual-level determinants whereas the CFIR focuses more on collective-level determinants [19]. The interview questions derived from relevant domains or constructs from CFIR + TDF (**Supplementary Material **1). The interviews were semi-structured following the interview guide but conducted as a conversation. The length of the interviews was 23 min on average. We discontinued collecting new data once we reached thematic saturation.

To determine the relevant CFIR + TDF domains/constructs, members of the research team who collectively represented expertise in urologic clinical care (MEN), implementation science (SAB), and practice-integrated health services research (JEL, LPS) were involved. For each selected domain and construct, questions were developed. During this process, we combined certain CFIR constructs and TDF domains that represented overlapping concepts. For example, ‘Self-efficacy’ (CFIR construct) and ‘Beliefs about capabilities’ (TDF domain) were combined since both focused on exploring providers’ confidence in their abilities to care for patients with PCa (or alternatively, AMH). To ensure adequate coverage and accurate representation of all relevant domains, two pilot interviews were conducted with practicing urologists. After the pilot interviews, interviewees provided feedback to the interviewer (LPS), particularly on question prompts and the overall flow of the interview.

### Analysis

Survey data were analyzed using descriptive statistics. We used Fisher’s exact test to examine whether there were differences between the survey respondents and the interview participants and found no significant difference. We have also examined the correlation between guideline-concordant care and provider characteristics (i.e., sex, type of degree, and experience) using Fisher’s exact tests but found no significant results. Interviews were audio recorded and transcribed verbatim. Transcripts were entered into MAXQDA qualitative software to facilitate data management and analysis. We used a directed content analysis approach [20] to code and analyze interview transcripts, beginning with *a priori* codes based on CFIR + TDF constructs/domains (**Supplementary Material **2). We coded any utterances related to the imaging decision-making process in the relevant CFIR + TDF construct/domain. For each utterance, we also identified whether the relevant construct/domain was specific to PCa or AMH imaging. Two researchers (SH, LPS) independently coded all transcripts and then discussed and reviewed the coding of each transcript to reconcile any coding discrepancies. If consensus could not be reached, they consulted a third reviewer (SAB), who has expertise in organizational theories, models, and frameworks. For each clinical condition, we identified relevant constructs/domains based on the frequencies with which the construct/domain was mentioned across interviews. Then, among each relevant construct/domain, we identified subthemes that could be either a single response or collection of responses from interviews illustrating similar opinions or statements. We next examined subthemes similarities and differences between PCa and AMH. The qualitative findings were used to confirm and provide additional detail for the quantitative survey results, adding a narrative understanding of the imaging behavior of clinicians for both PCa and AMH.

## Results

### Characteristics of participants

Out of 60 eligible respondents, 33 had completed the survey (55% response rate). Among the 10 respondents who expressed initial interest, 7 had agreed to participate in a telephone interview. The majority of survey respondents were male (n = 26, 79%) and had an MD or DO degree (n = 28, 85%). Most clinicians (n = 14, 42%) graduated with their degree (i.e. MD, DO, PA, or NP) between 1991 and 2003 (Table 1).

**Table 1:** Demographics of clinicians surveyed (N = 33)

	N	%
Sex		
Male	26	79
Female	7	21
Degree		
MD/DO	28	85
NP/PA	5	15
Graduation Year		
1990 or before	7	21
1991–2003	14	42
2004 or after	12	36

Abbreviations: Doctorate of Medicine/Doctorate of Osteopathic Medicine (MD/DO); Nurse Practitioner/Physician Assistant (NP/PA)

### Survey results

Table 2 provides the vignettes and results from the structured survey. For the first PCa patient (Patient 1), a 67-year-old otherwise healthy male newly diagnosed with low-risk PCa, all clinicians’ answers were concordant with guideline recommendations [18] except for one respondent who indicated they would use a different approach if the patient was interested in active surveillance. For Patient 2, a 64-year-old otherwise healthy, biological male newly diagnosed with low-intermediate-risk PCa, 6% of respondents reported they would use a CT scan or bone scan, which was guideline-discordant. For intermediate-risk PCa (Patient 3) and high-risk PCa (Patient 4) patients, where the clinical guidelines do recommend imaging (i.e., MRI, CT scan, and/or bone scan), 15% and 6% of respondents, respectively, reported they would not use imaging.

**Table 2:** Survey respondents answers to imaging scenarios (N = 33)

Imaging for PCa Patients		
**Patient 1a**: 67 year old otherwise healthy man newly diagnosed with low-risk prostate cancer (PSA level 5.2ng/ml, Gleason score 6 in 5/12 cores, clinical stage T1c, prostate volume 46 cc on TRUS).		
	n	%
No imaging (GL concordant)	30	91%
MRI (GL concordant)	3	9%
**Patient 1b**: After reviewing treatment options, the patient described above (PSA level 5.2ng/ml, Gleason 6 in 5/12 cores, clinical stage T1c) is interested in active surveillance.		
	n	%
No imaging (GL concordant)	21	64%
MRI (GL concordant)	11	33%
CT scan	1	3%
**Patient 2**: 64 year old otherwise healthy man newly diagnosed with low-intermediate-risk prostate cancer (PSA level 9.2ng/ml, Gleason score 3 + 4 in 3/12 cores, Gleason 6 in 2/12 cores; clinical stage T1c, prostate volume 53 cc on TRUS).		
	n	%
No imaging (GL concordant)	24	75%
MRI (GL concordant)	6	19%
CT scan or bone scan	2	6%
**Patient 3**: 69 year old otherwise healthy man newly diagnosed with high-intermediate-risk prostate cancer (PSA level 11.1ng/ml, Gleason score 4 + 3 in 4/12 cores, Gleason 3 + 4 in in 2/12 cores; clinical stage T1c, prostate volume 47 cc on TRUS)		
	n	%
No imaging	5	15%
MRI or CT or bone scan (GL concordant)	28	85%
**Patient 4**: 66 year old otherwise healthy man newly diagnosed with high-risk localized prostate cancer (PSA level 16.2ng/ml, Gleason score 4 + 4 in 5/12 cores, Gleason 4 + 3 in in 2/12 cores; clinical stage T2a, prostate volume 62 cc on TRUS)		
	n	%
No imaging	2	6%
MRI or CT or bone scan (GL concordant)	31	94%
**Imaging for AMH Patients**		
**Patient 5**: 37 year old nonsmoking female with no significant past medical history referred to you for asymptomatic microscopic hematuria (7 erythrocytes/HPF on microscopic urinalysis). Urine culture negative.		
	n	%
No imaging	3	9%
Renal ultrasound	11	33%
CT urogram or flexible cystoscopy (GL concordant)	12	36%
Repeat urinalysis*	7	21%
**Patient 6**: 40 year old nonsmoking male with no significant past medical history referred to you for asymptomatic microscopic hematuria (> 3 erythrocytes/HPF on two separate microscopic urinalysis). Urine culture negative.		
	n	%
No imaging	5	15%
Renal ultrasound	5	15%
CT urogram or flexible cystoscopy (GL concordant)	23	70%
**Patient 7**: 62 year old nonsmoking female with no significant past medical history referred to you for asymptomatic microscopic hematuria (> 3 erythrocytes/HPF on one microscopic urinalysis). Urine culture negative.		
	n	%
No imaging	5	15%
Renal ultrasound	7	21%
CT urogram or flexible cystoscopy (GL concordant)	16	48%
Repeat urinalysis*	5	15%

Abbreviations: GL, guideline

GL concordant: For AMH, guideline concordant is based on the AUA guidelines in 2020.

*Write in response

For all AMH patients (Patients 5–7 with different demographics and number of urinalyses), the AUA guideline at the time of the study period recommended that patients with AMH receive a CT urogram or flexible cystoscopy [14]. However, if a patient had received only one urinalysis (Patients 5 and 7), 15–21% of respondents wrote in that they would first repeat the urinalysis test. Only 36% of respondents reported they would use CT urogram or flexible cystoscopy for a 37-year-old, nonsmoking female with no significant past medical history (Patient 5) which was guideline concordant and only 48% reported doing so for a 62-year-old, nonsmoking female with no significant past medical history (Patient 7). However, for the vignette describing a patient who had > 3 erythrocytes/high power field on two separate microscopic urinalyses (Patient 6), 70% of respondents reported they would use a CT urogram or flexible cystoscopy which was guideline concordant.

### Semi-structured interview results

Multilevel determinants at the individual (patient and provider), organization/practice, and the external/policy levels were identified as critical to image decision-making. Within these multilevel determinants, we identified five common subthemes across both PCa and AMH that played a role in clinicians’ decision-making process (Table 3**)**: National Guidelines, Supporting Evidence and Information Exchange, Organization of the Imaging Pathways, Patients’ Clinical and Other Risk Factors, and Clinicians’ Beliefs and Experiences Regarding Imaging.

**Table 3:** Quotes for each subtheme illustrating similarities and differences between prostate cancer (PCa) and asymptomatic microscopic hematuria (AMH)

	Representative quotes related to PCa	Representative quotes relate to AMH
***External/Policy Level: National Guidelines***		
Follow guidelines (PCa) vs. “guideline-based” (AMH)	“The NCCN guidelines. Most guidelines suggest imaging for only high risk prostate cancer patients. So I follow those.” (*Participant 2)*	“…And I’m trying to think, --yeah, kind of based off the guidelines. How much blood are they seeing in the urine? If it’s an initial patient that comes in, you know, and they’re over the age of 35, they’ve had no work up whatsoever, I image them. If they’ve had previous microscopic hematuria work ups in the past, a lot of times my question is kind of how long ago have they had some imaging. And guidelines right now say to repeat the work up every three to five years.” (*Participant 23*)
High quality PCa guidelines vs. low quality AMH guidelines	“Sometimes in evaluating the guidelines that the bulk of AUA guidelines are made up from urologists. NCCN guidelines do I sometimes feel have a slightly more balanced view, and they have primary care providers, radiation oncologists, medical oncologists, urologists, statisticians, epidemiologists on their guideline committees.” (*Participant 15)*	“And I know that most of the guidelines are like Grade C evidence or the expert opinion for microscopic hematuria. So they’re not great guidelines, but from my perspective I feel like that’s what I’ve got to work with.” (*Participant 13)*
***External/Policy Level: Supporting Evidence and Information Exchange***		
Information exchange (PCa) vs. no information exchange (AMH)	“I think most of us are alerted to our professional again through AUA organization. We do talk about patient evaluations constantly with the residents and with each other. So there’s some word of mouth certainly involved.” (*Participant 36*)	“I’d say I honestly don’t know because I just see what I do. That I don’t know what other people are doing.” (*Participant 2*)
Literature not supporting imaging	“Just read the literature. Read the literature. If you’re treated for prostate cancer, you’ll more likely die younger than those who aren’t treated for prostate cancer.” (*Participant 4)*	“I know there’s literature out there that says an ultrasound is going to find 98% of anything wrong. The risk of bladder cancer is about zero. So I kind of handle it like that.” (*Participant 4)*
***Organization/Practice Level: Organization of the Imaging Pathways***		
Pre-appointment imaging	“Yeah. The way that our practice is set up is such that anybody in multidisciplinary clinic is where our new prostate cancer patients come through. The schedulers already know what the criteria are. And so they will order the appropriate tests. And I very rarely have to direct any of that. It just happens naturally based upon our algorithm.” (*Participant 48)*	“And in our health system, most primary care physicians are the ones that are finding the microscopic hematuria. And our kind of best practice is that they already get imaging for them sent to us.” (*Participant 36)*
Outside institution imaging	“If they’re a patient that’s referred from an outside institution, we may not actually have the images to view, which is sometimes challenging. We’ll have to request them, and sometimes that delays care. Or we’ll just have a report of our outside radiologist’s interpretation without being able to look at the images ourselves to confirm that we agree with that radiologist. So that sometimes delays care. But if those two scenarios are the case, then we obtain that imaging from whatever center did it, send it to us, and then we review it at our institution.” (*Participant 15)*	--
Electronic health record systems	--	“I think it’s, --well, to make it a little more convoluted, when we used the electronic note templates that we have set up, we have one that we use in our department for asymptomatic microscopic hematuria. And so we just plug in that template and there’s a drop down menu saying CT urogram according to AUA guidelines, or a drop down menu renal ultrasound…. But you can do drop down menus saying, okay, I’m getting a CT urogram in accordance with the AUA guidelines, or you can say this patient has renal insufficiency and cannot tolerate a CT urogram. I’m obtaining them a renal ultrasound.“ (*Participant 15)*
***Individual/Patient Level: Patients’ Clinical and Risk Factors***		
Patient clinical/ sociodemographic factors	“Well, I have a lot of old patients. So, I look at the bulk, the grade, physical exam, the age of the patient, and the PSA.“ (*Participant 4)*	“And so patients that are older, definitely I image. Smokers I image. If they’ve had a history of stones and they haven’t had any recent imaging, sometimes I’ll image them.“ (*Participant 23)*
“Risk-based approach” (AMH)	--	“I’m just assuming that probably some people are very more strictly adhering to the guidelines and then others like myself do a little bit more of a risk based approach.“ (*Participant 2)*
Reconfirm AMH diagnosis	--	“…If they have asymptomatic microscopic hematuria, the first thing I do is double check that that’s actually what they have because most people have a dipstick and it’s like one plus urine…. So I would just say that once I’ve confirmed that, greater than three red blood cells per high power field then I will move on to the workup…” (*Participant 2)*
Counseling patients on imaging	“I usually really use the score of the digital rectal exam and PSA to determine whether they get imaged or not, not based on whether a patient wants the image or really wants to skip imaging. I try to counsel them on the need when it’s appropriate.“ (*Participant 2)*	“One thing I have learned over the years to do is when I counsel patients upfront about hematuria, I proactively tell them that we are not here to explain every case of hematuria because that’s one thing the patients often want to know is like, “Well, why, because I’ve been told this is like this abnormal thing. I feel like I should understand why.” I proactively counsel them, “Well, that’s actually not the goal with hematuria workup because the data are that we’ll find maybe tops, one out of four, we’ll be able to attribute the hematuria to something. But for most people, we can’t find that reliable cause for it,” so yeah. (*Participant 48)*
***Individual/Provider Level: Clinicians’ Beliefs and Experiences Regarding Imaging***		
Imaging does not improve patient care	“I feel like the nomograms because it allows us to say, “You have a 1% chance of having any cancer in your lymph nodes, so it just makes no sense to image a node. You have essentially a negligible chance, somewhere between zero and 0.001 chance, of having cancer in your bones, so we just should not do this.” (*Participant 48)*	“If you came in here and you had microscopic hematuria and no symptoms whatsoever, and let’s say, --we do dipsticks, so small to a trace, I would tell you, --and you don’t smoke. You don’t have any symptoms. And I’d say, yeah, there’s probably less than 1% chance you having anything bad. Does that bother you? And people say, “What? How low?” Less than 1%….So it’s a very low risk.“ (*Participant 4)*
Legal protection by following guidelines (AMH)	--	“… certainly the medical/legal environment, I think, also is I think something that is always in the back of people’s minds here in the United States. It’s hard to quantify how much that risk is because I think the system tends to be somewhat arbitrary and precious, and so it’s kind of hard to predict. Maybe that makes it even worse since it is so hard to predict what is and is not an exposure and then you’re maybe best served by being as cautious as you can going right by the guidelines.“ (*Participant 48)*

#### External/Policy level: National Guidelines

Participants were aware that there were national guidelines for imaging patients with PCa and AMH. For AMH, participants specifically referred to the AUA guidelines. Both AUA and National Comprehensive Cancer Network (NCCN) guidelines were mentioned for imaging PCa patients:

“I would probably look first to the AUA. I think they have guidelines for localized prostate cancer, or I would look at the National Comprehensive Cancer or the National Cancer Center Network, NCCN. I think that’s the other source of guidelines that I typically go to.” (*Participant 48)*

Some participants acknowledged that they were more likely to follow NCCN guidelines since the AUA guidelines were more difficult to access and to understand:

“Often I have to log in to the AUA website sometimes and I can’t remember my user ID and password…. NCCN usually has a fairly easy to understand algorithm that you can just kind of follow the boxes on what to do.” *(Participant 15)*

Clinicians indicated that they were likely to systematically follow guidelines for imaging PCa patients. On the other hand, their comments regarding the following of guidelines for imaging use among AMH patients, were more nuanced. While some clinicians systematically followed the guidelines, which at the time of the interview recommended CT for all, most reported following more of a risk-based approach for AMH imaging, a direction in which the field has since moved, with an updated guideline released after the study period. This variability among clinicians in guideline concordance was attributed to the perceived low-quality evidence supporting the previous AMH guideline by the respondents. One clinician mentioned:

“most of the guidelines are like Grade C evidence or the expert opinion for microscopic hematuria. So they’re not great guidelines.” *(Participant 13)*

Additionally, clinicians often felt that the AMH guidelines did not give them enough information to make an informed imaging decision whereas guidelines for PCa imaging were praised for being up-to-date and clear:

“there’s not a lot of clear-cut guidelines in patients that have stones, kind of what you do with those patients.” *(Participant 23)*

#### External/Policy level: supporting evidence and Information Exchange

Clinicians sought information from medical literature that would inform their PCa and AMH imaging decision-making. Often, clinicians referenced the literature in favor of less imaging for both PCa and AMH. For PCa, a clinician stated:

“…so I’m usually pretty firm with folks about why I don’t think it’s helpful and what the probability is. I feel like the nomograms…allows us to say, “You have a 1% chance of having any cancer in your lymph nodes, so it just makes no sense to [image] a node. You have essentially a negligible chance, somewhere between zero and 0.001 chance, of having cancer in your bones, so we just should not do this.”” *(Participant 48)*

Similarly, for AMH, another clinician stated,

“But when we see repeated, repeated, repeated papers…that catch a lot of headlines saying, hey, we can reduce the radiation and just get an ultrasound…” (*Participant 15)*

Medical literature seemed to serve as a confirmation that imaging for AMH was often not beneficial to patients. However, one clinician also cited literature as a reason for creating a direct pathway for imaging AMH patients:

“there are certainly papers that have been published about doing these kinds of things where you streamline the hematuria pathway.” *(Participant 48)*

Clinicians also mentioned seeking information that would inform their decision-making for PCa imaging through other formal and informal avenues. For example, clinicians mentioned learning about updated guidelines information through emails and even social media:

“I just generally use the guidelines. I’ve seen information on Twitter [and] many places.” (*Participant 2)*

#### Organization/Practice Level: Organization of the imaging pathways

For PCa, most clinicians discussed that patients were often imaged before their scheduled appointment through streamlined pathways:

“The schedulers already know what the [PCa imaging] criteria are. And so they will order the appropriate tests. And I very rarely have to direct any of that. It just happens naturally based upon our algorithm.” *(Participant 2)*

While this type of streamlined pathway potentially ensured that guideline-concordant imaging was used, clinicians also mentioned that patients would also be imaged, both inappropriately and appropriately, prior to their scheduled appointment if they had first gone to another institution. One clinician explained:

“If they’re a patient that’s referred from an outside institution, we may not actually have the images to view, which is sometimes challenging. We’ll have to request them…. Or we’ll just have a report of our outside radiologist’s interpretation without being able to look at the images ourselves to confirm that we agree with that radiologist…. But if those two scenarios are the case, then we obtain that imaging from whatever center did it, send it to us, and then we review it at our institution.” *(Participant 15)*

For AMH, there was more variability in the contexts in which imaging was ordered. Some clinicians mentioned that it was a patient’s primary care provider who would order imaging for AMH:

“In our health system, more primary care physicians are the ones that are finding the microscopic hematuria. And our kind of best practice is that they [the primary care providers] already get imaging for them [the patients] sent to us.” *(Participant 36)*

However, in contrast to PCa, specialist clinicians had more discretion in imaging decision-making for AMH:

“I would order this [imaging] myself. It’s more individualized.” (*Participant 2)*

One clinician mentioned how electronic health record (EHR) systems played a role in their imaging decision process; specifically, the EHR consisted of the template with a drop-down menu of CT urogram based on the AUA guidelines. If clinicians did not choose a CT urogram from the drop-down menu, then they would be asked to document the reason for not choosing the guideline-concordant option.

#### Individual/Patient level: patients’ clinical and other Risk factors

For both PCa and AMH, participants consistently mentioned considering both patients’ clinical and other risk factors in their image decision-making process. Specifically, for PCa, clinicians mentioned clinical factors such as PSA and Gleason score. Once these clinical factors were known, clinicians felt they could easily follow the guidelines. In contrast, clinicians were more likely to take a risk-stratified approach for AMH patients, which would involve considering patients’ sociodemographic factors, particularly patient’s age, smoking behavior, prior exposure to radiation, and their field of work (e.g., occupational exposures to carcinogens).

One common clinical process stood out for AMH imaging; clinicians consistently mentioned that before they put their patients through imaging, they would run another urinalysis to confirm the results:

“I will for sure get them a second urinalysis to confirm that they truly have asymptomatic microscopic hematuria. I won’t pull the trigger on imaging and cystoscopy until we’ve had more than one urinalysis showing that.” *(Participant 15)*

For both AMH and PCa, many clinicians raised the importance of counseling patients on why imaging was or was not recommended. When seeing patients with AMH, proactive discussions were integral to helping patients understand the rationale for imaging (or not imaging):

“One thing I have learned over the years to do is when I counsel patients upfront about hematuria, I proactively tell them that we are not here to explain every case of hematuria because that’s one thing the patients often want to know is like, ‘Well, why, because I’ve been told this is like this abnormal thing. I feel like I should understand why.” I proactively counsel them, “Well, that’s actually not the goal with hematuria workup because the data are that we’ll find maybe tops, one out of four, we’ll be able to attribute the hematuria to something. But for most people, we can’t find that reliable cause for it.” *(Participant 48)*

For PCa, clinicians talked about making imaging decisions based on objective clinical assessments and relying less on a patient’s preference:

“I usually really use the score of the digital rectal exam and PSA to determine whether they get imaged or not, not based on whether a patient wants the image or really wants to skip imaging. I try to counsel them on the need when it’s appropriate.” *(Participant 15)*

#### Individual/Provider level: Clinicians’ beliefs and experiences regarding imaging

Clinicians mentioned that the anticipated consequences of imaging played a role in their decision-making process. For both low-risk PCa patients and AMH patients, clinicians felt that they would often decide not to image a patient because it would not directly benefit patient outcomes. With respect to PCa, clinicians explained:

“And I know the chance of dying from disease itself is very low for most of these things.” (*Participant 4*)

“…so we don’t necessarily have great data that [imaging] improves patient outcomes.” (*Participant 23*)

Similarly, for AMH patients, clinicians asserted:

“…so I think the problem, there is certainly radiation exposure to the patients, costs to the patient and the healthcare system. I think those are the two biggest things. There is some potential harm from exposure to contrast, which is typically part of the guideline concordant evaluation for hematuria.” (*Participant 2*)

“If you came in here and you had microscopic hematuria and no symptoms whatsoever, and let’s say, --we do dipsticks, so small to a trace, I would tell you, --and you don’t smoke. You don’t have any symptoms. And I’d say, yeah, there’s probably less than one percent chance you having anything bad.” (*Participant 4*)

Furthermore, participants often brought up how imaging could result in incidental findings instead of useful information regarding the patient’s current condition. Clinicians frequently explained that imaging for low-risk PCa and AMH also led to a variety of negative consequences for patients as well as higher medical costs. Among AMH patients in particular, one clinician mentioned:

“Effectively, we’d have to put them through cystoscopy, which can be uncomfortable and also have a number of downstream effects such as urinary tract infection and recurrent bleeding and things like that.” *(Participant 15)*

Despite acknowledging such detrimental consequences, for AMH, clinicians reported ordering imaging to avoid legal consequences:

“…I mean you’re setting yourself up from a liability standpoint [for practicing outside the guidelines].” *(Participant 36)*

## Discussion

Findings from the survey and interview collectively contributed to uncovering the complexity of de-implementation in urology care in that multilevel determinants influenced clinicians’ imaging behavior for PCa and AMH. The survey results showed that PCa scenarios overall had higher guideline-concordance than among the AMH scenarios. Interview data further informed that determinants at the individual level (patient and provider), organization/practice level, and the external/policy level were strong determinants of urologists’ imaging behavior for both clinical conditions. While the identified themes across these multilevel constructs were similar across clinical conditions, how the multilevel determinants were contextualized often differed by clinical condition. These mixed methods findings complement previous studies that documented the quantitative prevalence of low-value imaging [1, 2]. Our results imply that multilevel interventions for de-implementing LVC need to be developed and that those interventions are likely best tailored to address the determinants that are specific to each clinical condition. Our approach also builds upon previous imaging and implementation literature [21] by identifying determinants of imaging across two distinct clinical conditions, which provided the opportunity to compare and contrast the extent to which multilevel factors beyond a patient’s condition drive a clinician’s imaging behavior.

Findings on the multilevel determinants of imaging for PCa and AMH suggest potential strategies [22] that could be leveraged when trying to de-implement low-value imaging practices and also to promote appropriate imaging when necessary. The decision-making criteria for PCa imaging was perceived as relatively straightforward and the consensus among study participants was to follow existing clinical practice guidelines; however, studies have shown that intent does not always translate into action (i.e., following the guidelines) which was also verified from our survey results. In fact, in some instances, providing information on the guidelines has reduced imaging, even in scenarios where imaging was appropriate [23]. Thus, based on our evidence that clinicians referred to medical literature, guidelines may benefit from accompanying educational materials (e.g., appropriate scenarios and examples, medical literature citations). Augmenting guidelines with evidence and examples may facilitate guideline-concordant imaging practices. Additionally, using an audit and feedback strategy through which clinicians are given personalized reports of their imaging practices may be useful as shown in other previous studies on improving guideline adherence [24, 25]. This would allow clinicians to know to what extent they were following the guidelines and ensure that their intention and practice were aligned.

Compared to PCa, the context of imaging for AMH was more dynamic in that clinicians’ decisions ranged from systematically following the guidelines to taking an idiosyncratic risk-stratified approach. In the period of this study, the existing 2012 guideline recommended intensive, one-size-fits-all CT imaging [14]; however, emergent research questioning this approach led to a revision of the guideline [13] after the study period which provided clearer guidance and patient-centered approach. Taking into account this additional context, our finding that some clinicians were less likely to strictly follow the AMH guidelines may be perceived as a positive gradual movement towards reducing low-value imaging. In the absence of strong evidence supporting the AMH guidelines and the identified lack of communication among clinicians on their AMH imaging practices, promoting network weaving which builds on existing working relationships and networks outside the organizational boundary or creating a collaborative among organizations to foster an environment of learning and communication may be an effective de-implementation strategy since it would improve the consistency and appropriateness of imaging behavior within and across practices. For instance, MUSIC (Michigan Urological Surgery Improvement Collaborative), a statewide quality improvement collaborative comprising 42 diverse practices, has demonstrated success with improving imaging utilization using multidimensional interventions that involved education, site visits/monitoring, development of scripts [26]. In particular, it may be beneficial to develop collaboratives between academic medical institutions, where the latest research is being conducted, and non-academic organizations, where guideline updates are often less than timely. Lastly, for AMH, there was more variability in the clinical pathways and people involved in image ordering. Strategies such as developing a quality monitoring system would enable practices to assess through clinical pathways and determine at which step low-value imaging is occurring. Next, developing tools in their system to further prevent unnecessary imaging within the organization is needed, such as through EHR prompts and alerts which had been proven to be successful strategies in changing providers’ awareness and behavior [27–29].

Using the example of imaging in both PCa and AMH, this study used existing implementation frameworks to comprehensively explore the multilevel determinants of imaging and examine how these determinants varied across clinical conditions [17]. This approach enabled a more systematic assessment of multilevel determinants of imaging, including its contextual determinants for de-implementation. Despite being crucial to identifying and developing effective de-implementation strategies, contextual determinants are often are overlooked in studies that focus on identifying determinants of, specifically, low-value imaging [30]. Moreover, we examined imaging in two different clinical conditions in which low-value imaging is common and investigated the similarities and differences of the determinants in different disease contexts. This facilitated the opportunity to examine the extent to which multilevel factors beyond the type of clinical condition affect a clinician’s imaging behavior.

This study had a number of limitations. Although the study combined surveys with semi-structured interviews, generalizability may be limited due to the small number of interviewees. However, based on participants’ demographic information (i.e., sex, type of degree, experience), interview participants did not differ from surveyed clinicians. Secondly, our study focused exclusively on potential determinants of ordering imaging among urologists and urology-affiliated clinicians. We did not include primary care providers who may also evaluate AMH patients. Participants were from academic medical centers and academically-affiliated healthcare systems, excluding perspectives from independent private practice settings. This may also impact the generalizability of the findings to other clinical settings. Lastly, while we suggested some de-implementation strategies based on the identified determinants of imaging, future research could also use a more systematic approach, such as implementation mapping, to link determinants to a set of de-implementation strategies [31].

## Conclusion

Findings from the study showed de-implementation of low-value imaging requires understanding of the multilevel determinants of imaging and the specific nuances of the clinical contexts. Results will inform future implementation mapping efforts to develop low-value imaging de-implementation strategies with identified multilevel determinants which is part of the implementation mapping process. Further research is needed to evaluate whether determinant-specific de-implementation strategies are effective in reducing low-value imaging and to understand if and how their effectiveness varies by context. Also, future work should be conducted on other LVC to see whether any of the identified level of determinants are generalizable to other types of care. Through these efforts, we will take further steps forward to unpacking the complexity of de-implementation in urology care.

## Electronic supplementary material

Below is the link to the electronic supplementary material.

Supplementary Material 1

Supplementary Material 2

## Data Availability

• The datasets generated during the current study are not publicly available due to the confidentiality afforded study participants, but are available in limited, redacted form from the corresponding author on reasonable request.

## Abbreviations

LVC: Low-Value Care.
AMH: Asymptomatic Microscopic Hematuria.
PCa: Prostate Cancer
CFIR: Consolidated Framework for Implementation Research
TDF: Theoretical Domains Framework
MRI: Magnetic Resonance Imaging
CT: Computed Tomography
PSA: Prostate Specific Antigen
AUA: American Urological Association
ASCO: American Society of Clinical Oncology
NCCN: National Comprehensive Care Network
EHR: Electronic Health Record
