# Supplementary material for: Understanding the multilevel determinants of clinicians’ imaging decision-making: setting the stage for de-implementation of low-value imaging
Source: BMC Health Serv Res. 2022 Oct 5;22:1232. doi: 10.1186/s12913-022-08600-3 (PMC9535949; doi:10.1186/s12913-022-08600-3)
Supplement: Supplementary file 1 — Supplementary Material 1 [file 12913_2022_8600_MOESM1_ESM.docx]

**Appendix 1. Interview Guide**

***NOTES TO INTERVIEWER:***

The goal of this interview guide is to *understand how you make decisions on imaging patients with prostate cancer and patients with asymptomatic microscopic hematuria*

**Introduction:**

Thanks Dr. XXX for participating in this interview. Is this still a good time for you?

The purpose of this interview is to help us understand how you decide whether to image patients with two common urological conditions, prostate cancer and asymptomatic microscopic hematuria. Our discussion should last about 20-25 minutes. All of your responses will remain confidential and reported in aggregate. You may choose to stop the interview at any time, and there is no penalty to you for not completing the interview.

Before we begin, I just wanted to ask if it is ok if I record our discussion for the purposes of transcription? Your name or any personal identifiers will not be associated with any of the notes. The audio recordings will be deleted once the project is complete.

Do you have any questions before we begin?

1. **Let’s begin the interview then. Do you see these patients with either AMH or prostate cancer in your practice? Which do you see more often? Ok, let’s start our discussion by talking about [prostate or AMH] patients.**
2. **Starting with prostate cancer patients, would you walk me through how you decide whether or not to image a prostate cancer patient? What is your thought process or reasons for imaging or not imaging a patient with prostate cancer.**

***NOTE TO INTERVIEWER: Below, is a checklist of TDF domains and CFIR constructs, with example prompts for each. As participant responds to above questions, using the blank column to the right of each diagnosis (i.e. “prostate cancer” and “AMH”) column, check off items that they address. After the participant responds, prompt them to address any checklist item which they haven’t already addressed. You don’t necessarily need to ask every single prompt as listed, but you should address each listed domain/construct.***

***NOTE TO INTERVIEWER: Once you have covered prostate cancer, then switch to imaging for patients with AMH.***

1. **Thanks for providing that information. Let’s move on to discussing what happens when you are presented with a patient with AMH. Would you walk me through how you would decide to image a patient with AMH?**

| **CFIR: OUTER SETTING** | | |  |  |  |
| --- | --- | --- | --- | --- | --- |
|  | | **PROSTATE CANCER** | **Indicate if Theme Covered with “X”** | **AMH** | **Indicate if Theme Covered with “X”** |
|  | **Patient needs and resources** | Do you think patients with prostate cancer prefer to be imaged? (*Prompts: liability concerns*) |  | Do you think patients with AMH cancer prefer to be imaged? (*Prompts: liability concerns)* |  |
|  | **Cosmopolitanism** | How do you exchange information with other physicians regarding prostate cancer imaging outside of your local practice? |  | How do you exchange information with other physicians regarding AMH imaging outside of your local practice? |  |
|  | **Peer pressure** | To what extent do you think your colleagues in other practices image their prostate cancer patients? |  | To what extent do you think your colleagues in other practices image their patients with AMH? |  |
|  | **External policy & incentives** | Are there any local or national guidelines that play a role in whether or not you image a prostate cancer patient? |  | Are there any local or national guidelines that play a role in whether or not you image a patient with AMH? |  |
| **CFIR: PROCESS** | | |  |  |  |
|  | **Engaging** | Are there key influential individuals that would affect whether you or clinicians in your organization/practice decide whether or not to image prostate cancer patients? |  | Are there key influential individuals that would affect whether you or clinicians in your organization/practice decide whether or not to image a patient with AMH? |  |
| **CFIR INNER SETTING** | | |  |  |  |
|  | **Structural characteristics/Environmental Context (TDF)** | Do you think the infrastructure of your organization (social architecture, age, maturity, size, or physical layout) affects how clinicians decide whether or not to image prostate cancer patients? (*Prompts: EMR alerts for best practices, pop-up notifications*) |  | Do you think the infrastructure of your organization (social architecture, age, maturity, size, or physical layout) affects how clinicians decide whether or not to image a patient with AMH? (*Prompts: EMR alerts for best practices, pop-up notifications*) |  |
|  | **Networks & communication** | How do you typically find out about new information within your organization/practice, such as new initiatives? |  | How do you typically find out about new information within your organization/practice, such as new initiatives? |  |
|  | **Culture** | Are there any aspects of your organization's culture (general beliefs, values, assumptions that people embrace) that affects whether or not you decide to image patients with prostate cancer? |  | Are there any aspects of your organization's culture (general beliefs, values, assumptions that people embrace) that affects whether or not you decide to image patients with AMH? |  |
|  | **Implementation climate** | Are there standard work processes and practices regarding imaging of prostate cancer patients in your practice? |  | Are there standard work processes and practices regarding imaging of patients with AMH in your practice? |  |
| **CFIR: INDIVIDUAL CHARACTERISTICS /**  **TDF DOMAINS** | | |  |  |  |
|  | **Knowledge (TDF)/ Knowledge and beliefs about the intervention (CFIR)** | Are there clinical guidelines that make recommendation regarding imaging for prostate cancer patients? |  | Are there clinical guidelines that make recommendation regarding imaging for patients with AMH? |  |
|  | **Beliefs about capabilities (TDF)/ Self-efficacy (CFIR)** | How confident are you in managing prostate cancer patients? (*Prompts: problems you may encounter/additional expertise or experience needed*) |  | How confident are you in managing patients with AMH? |  |
|  | **Beliefs about consequences (TDF)** | What are the potential benefits or disadvantages to not imaging a patient with prostate cancer? |  | What are the potential benefits or disadvantages to imaging a patient with AMH? |  |
|  | **Motivation & goals (TDF)** | Do you feel that it is important not to image prostate cancer patients? |  | Do you feel that it is important to image patients with AMH? |  |
|  | **Memory, attention, and decision processes (TDF)** | When presented with a patient with prostate cancer, what, if any, are the tasks that you do automatically? (*Prompts*: Would there be conditions under which you would consider imaging a prostate cancer patient an automatic task?) |  | When presented with a patient with AMH, what, if any, are the tasks that you do automatically? (*Prompts*: Would there be conditions under which you would consider not imaging a patient with AMH an automatic task?) |  |
|  | **Environmental context and resources (TDF)** | What physical or environmental resources would influence you not to image a prostate cancer patient?  (*Prompt: having onsite radiology equipment/staff to help/reimbursement/standing orders/order sets/pathways/speed with which results are available/patient’s insurance coverage?*) |  | What physical or environmental resources would influence you not to image a patient with AMH?  (*Prompt: having onsite radiology equipment/staff to help/reimbursement/standing orders/order sets/pathways/speed with which results are available/patient’s insurance coverage?*) |  |
|  | **Social Influences (TDF)/ Individual Identification with the organization (CFIR)** | How might views or opinions of others, such as colleagues, patients, professional groups, or others in your practice influence whether or not you image prostate cancer patients? |  | How might views or opinions of others, such as colleagues, patients, professional groups, or others in your practice influence whether or not you image patients with AMH? |  |
|  | **Emotion (TDF)** | How might patient emotions such as worry/concern influence whether or not you image prostate cancer patients? |  | How might patient emotions such as worry/concern influence whether or not you image patients with AMH? |  |
|  | **Behavioral regulation (TDF)** | What do you think would have to change at your practice to deter imaging of prostate cancer patients? |  | What do you think would have to change at your practice to encourage imaging of patients with AMH? |  |

Those are all of the questions that I had planned for today. Is there anything else that you want to tell me that would help me to understand your decision to image patients with AMH or prostate cancer?

Thank you for your time!
