# Supplementary material for: Understanding the multilevel determinants of clinicians’ imaging decision-making: setting the stage for de-implementation of low-value imaging
Source: BMC Health Serv Res. 2022 Oct 5;22:1232. doi: 10.1186/s12913-022-08600-3 (PMC9535949; doi:10.1186/s12913-022-08600-3)
Supplement: Supplementary file 2 — Supplementary Material 2 [file 12913_2022_8600_MOESM2_ESM.docx]

**Appendix.** Coding Guide (Analysis conducted using directed content analysis (Hsieh & Shannon, 2005))

Notes:

1. Conceptual frameworks are the Theoretical Domains Framework (Michie et al., 2005) and Consolidated Framework for Implementation Research (Damschroder et al., 2009).
2. Code domains, using constructs to justify the domain coding. See below for construct definitions.
   1. Make sure to identify whether provider is discussion prostate cancer imaging, AMH imaging, or both.
3. Make sure to code question as well as response when response does not reflect content of question.
4. Code both affirmative and negative responses.

|  | | | |
| --- | --- | --- | --- |
| **CFIR: OUTER SETTING** | | | **Examples**  ***(Note: Quotes exemplifying appropriate use of coding themes were included in codebook but removed for the Appendix to ensure interviewees’ identify was protected.)*** |
|  | **Patient needs and resources** | - Patient needs known and addressed by imaging/not imaging at an organizational-level or practice-level. - Does NOT refer to patient’s medical criteria (e.g., PSA, Gleason score, etc.) - Does NOT refer to patient’s needs being known and addressed by the provider. | Appropriate Use:  Inappropriate Use: |
|  | **Cosmopolitanism** | - Degree to which information regarding imaging is exchanged with other physicians outside of local practice (e.g., Professional networking engagement at conferences, through social media) | Appropriate Use:  Inappropriate Use: |
|  | **Peer pressure** | - Pressure to follow same imaging procedures as colleagues in other practices | Appropriate Use:  Inappropriate Use: |
|  | **Knowledge (TDF)/External policy & incentives (CFIR)** | - Knowledge and influence of local or national guidelines in imaging decision-making process - Financially-related incentives influencing imaging decision (e.g., report cards/dashboard). | Appropriate Use:  Inappropriate Use: |
| **CFIR: PROCESS** | | |  |
|  | **Engaging** | - Key influential individuals in the provider’s practice that affect provider’s imaging decision or his/her views/opinions of the guidelines (e.g., implementation leader or champion) | Appropriate Use:  Inappropriate Use: |
| **CFIR INNER SETTING** | | |  |
|  | **Structural characteristics (CFIR)/ Environmental context and resources (TDF)** | - Infrastructure of the organization (social architecture, age, maturity, size, or physical layout) influencing imaging decision (e.g., EMR alerts for best practices, pop-up notifications) - Physical and material resources for supporting (or not supporting) imaging (e.g., having onsite radiology equipment/staff to help/reimbursement/standing orders/order sets/pathways/speed with which results are available/patient’s insurance coverage) - The extent to which physical or resources factors facilitate or hinder imaging. | Appropriate Use:  Inappropriate Use: |
|  | **Networks & communication** | - Learning about new information regarding imaging within practice/organization - Sharing of information regarding new imaging guidelines/policies/practices within one’s organization. - Informal/formal sharing of information regarding image decision making   - Information could be specific to a particular patient or could refer to information about all patients | Appropriate Use:  Inappropriate Use: |
|  | **Social Influences (TDF)/Culture(CFIR)** | - Organizational culture (norms, general beliefs, values, assumptions) within practice regarding imaging - Consensus view/opinions of groups (e.g., colleagues/patients/professional groups/others)   - This can include groups or organizations that have influenced the provider in the past. - Note: compared to “Social role/identity (TDF)/ Individual Identification with the organization (CFIR),” this domain should be used when the identity/views of the group and not just the individual. | Appropriate Use:  Inappropriate Use: |
|  | **Implementation climate** | - Degree to which there is a need for change in current imaging practices - Capacity for change in one’s practice - Note: provider should refer to these processes at the practice-level (“we” do this) not the individual-level (“I” do this). | Appropriate Use:  Inappropriate Use: |
| **CFIR: INDIVIDUAL CHARACTERISTICS /**  **TDF DOMAINS** | | |  |
|  | **Beliefs about capabilities (TDF)/ Self-efficacy (CFIR)** | - Provider’s internal beliefs regarding his/her ability to care for patients and confidence in managing patients with or without imaging (despite additional problems that may be encountered). | Appropriate Use:  Inappropriate Use: |
|  | **Beliefs about consequences (TDF)** | - Consequences, or anticipated outcomes of imaging patients (or not imaging patients). Outcomes could be either benefits or harms from deciding to image or not image patients. | Appropriate Use:  Inappropriate Use: |
|  | **Motivation & goals (TDF)** | - Your intention or intrinsic motivation for imaging/not imaging patients – the provider’s priority in choosing whether or not to image a patient. | Appropriate Use: |
|  | **Memory, attention, and decision processes (TDF)** | - A provider’s personal automatic processes or his/her personal decision process for evaluating a patient for imaging. Additionally, conditions under which the clinician may choose the alternative. - Does not refer to automatic processes from clinics – only automatic processes done by provider. | Appropriate Use:  Inappropriate Use: |
|  | **Social role/identity (TDF)/ Individual Identification with the organization (CFIR)** | - Provider’s perceptions of attributes of the practice or role/identity within the practice that may affect the provider’s decision to image patients. - Note: compared to “Social influences/Culture,” this domain should be used when the identity/view specific to the individual and not the group. The identity/view of the individual is shaped or informed by membership in the group/practice. | Appropriate Use:  Inappropriate Use: |
|  | **Emotion (TDF)** | - Influence of patient’s emotion (worry/concern) or provider’s emotion on imaging decision. | Appropriate Use:  Inappropriate Use: |
|  | **Behavioral regulation (TDF)** | - Changes at practice that would influence imaging decision | Appropriate Use:  Inappropriate Use: |
